# Reflex Effects of Intranasal Disease in the Pharynx and Mouth1Read before the medical section, Buffalo Academy of Medicine, November 10, 1896.

**Published:** 1897-01

**Authors:** Horace Clark

**Affiliations:** Buffalo, 21 West North Street


### REFLEX EFFECTS OF INTRANASAL DISEASE IN THE PHARYNX AND MOUTH.[1]

By HORACE CLARK, A. B., M. D., of Buffalo.

IF I MAY be pardoned for a moment's pleasantry, I would suggest that the subject of reflexes is closely allied to that of mistaken diagnosis. It is our common experience to receive a case every few days that has been from one to the other seeking relief. The right thing is finally done. The patient wonders why he need have had so long a siege, and the fortunate physician or surgeon is apt to plume himself unduly upon his skill. It is, therefore, not irrelevant to take this opportunity to raise the question as to a twentieth century possibility of having a general distributing bureau for people who are ailing. It might be called "a high court of diagnosis." Each person would be provided with a blank form for reports upon the examination of the various organs, made by physicians and surgeons of acknowledged highest skill. These reports would be subject to the revision of an advisory board and the patient would be sent at once to the practising specialist outside, into whose hands the case would be most likely finally to fall. If success failed, then the next most salient feature in the case would be considered and disposed of in the same way. Such

1. Read before the medical section, Buffalo Academy of Medicine, November 10, 1896.

a method might lead to a "trust" of physicians and surgeons. I do not know but this would be a good thing. It would save the public from quackery and supply it with many persons who might be of signal use. It would be a short way of getting at the "survival of the fittest." That is what is bound to happen to each and every one of us eventually, but very often not until after years of struggle, with loss of means, pride and ambition.

A few cases of my own are illustrations. In the first case I made a hypothetical diagnosis of dysphagia, occasioned reflexly from extreme nasal hyperemia, combined with or followed by the same condition in the pharynx. The throat was greatly relaxed. I am constrained to think that cure resulted from treatment of the pathological conditions in the parts indicated.

Mrs. W. had been unable to swallow solid food for a dozen years. She was 35 years old when I saw her, of small stature and sanguine temperament. There was nothing about her manner, nor her history, as told by herself or her friends, to indicate hysteria. She had no opinion herself as to why she could not swallow properly. Several practitioners in neighboring towns had told her that there was organic disease of the esophagus, which occasioned obstruction, and she was finally sent to Dr. Wilcox, of this city, to be operated on. I was asked to see the case in consultation and advised examination under chloroform. Neither of us could get any sort of an instrument to penetrate more than three or four inches of the esophagus. Anesthesia had not been completed to our satisfaction, and it was agreed that I should attempt a diagnosis under cocaine. Failing in this, the first proposition would be repeated and we would be prepared to do an esophagotomy.

I used metal bougies. The first one tried was about one-quarter the size of an ordinary olive. Its passage was firmly resisted at a depth of about three inches. I allowed it to remain, however, and upon gentle urging it soon became tightly grasped. In the course of half an hour it had passed through to the stomach and back again, meeting the same obstruction at the same place on withdrawal and overcoming it in the same way. The following day the same instrument passed more readily and without cocaine. A few days later I cocainised the constricted part with a sound and catheter, and passed the next larger bougie. Repetition of this procedure from time to time resulted in the ready admission and passage of the largest bougie. Meantime, I discovered that irritation with the probe of certain portions of the inferior turbinated bodies, notably the left one, gave rise to gagging efforts when the mouth was held wide open, which were not present upon irritation of other parts. This irritable tissue was destroyed with the galvano-cautery, which was followed, in due time, by cessation of the gagging upon probing the parts operated. Involuntary swallowing was greatly reduced after the removal of degenerated tonsils with the cautery loop. The remainder of the treatment consisted of the daily application of the faradic current, alternately, to the fauces and esophagus, with the exhibition of strychnia and a laxative. Maltine with coca wine was added to a carefully selected dietary.

The point in the case is that the patient gradually came to a diet of solid food, which was taken without any difficulty whatever, and the result has continued uninterruptedly for nearly a year. Moreover, she has had but one cold in the head during that time, whereas previously she had been a sufferer in this way.

The second case is also one of dysphagia, but widely different in detail.

The patient is a young man, 19 years old, of good family and with all the surroundings of luxury. He is well built and strong, though undersized. I do not know when I have met a boy who understands himself as well, or has more of an executive, deliberate manner. He gave me his entire confidence, and has never made the slightest contradiction upon repeated cross-examination. He was sent to me for treatment of nasal catarrh. The dysphagia was looked upon as a spasmodic affection, due, probably, to masturbation. No positive evidence to support this etiology was possessed, and I was asked to try to obtain it. To my mind the case did not present such a picture, nevertheless most careful inquiry and examination was made to that end. The only other general cause for spasmodic dysphagia which could be drawn from the patient's history, was pin worms. These he has had, from time to time, since childhood. On the supposition that there might be other varieties of intestinal parasites, full doses of santonine were given, with careful directions to examine the dejections. Solid food is never regurgitated, nor is there often regurgitation of liquids. A bolus of food is passed on into the esophagus, but sticks, entire or in part, a little beyond the entrance. Difficulty in swallowing began as far back as the patient can remember. There has never been a remission or exacerbation, but the trouble has very gradually increased. At ten years of age he had so-called typho-malarial fever. He was very ill, and convalescence was protracted. When he was sixteen years old he struck on his head when diving. For a long time thereafter he could not take a complete inspiration. He states that his diaphragm seemed to be fixed at a certain place. This condition still pertains, but has been largely overcome by expansion of breathing surface from growth, and because the apices of the lungs have been put to greater use. The history relates, also, to tardy beginning and ending of micturition. The bowels are sluggish, although there is no constipation. Appetite and digestion are good, and there has been a gain in weight of ten pounds within the past two months. The eye on the side corresponding with the paretic side of the throat, presently to be mentioned, is abnormal in some way as to sight, and its conjunctiva inflames more readily than that of the other eye. The tuning-fork applied to the top of the head is heard more distinctly on the paretic side. The pulse has always been rapid, because of this last part of the history, and to make a test case, as far as possible, as to the causative relationship between nasal disease and dysphagia, Dr. Krauss was asked to make a careful examination. He reported an etiology of functional nervous origin entirely. There is no reason why a multiplicity of complications might not be coincident with the dysphagia, yet independent of it. I question if general neurosis is responsible for the dysphagia in this instance, preferring a diagnosis of local myopathic paresis. This proposition is already sufficiently sustained, by radical improvement, subjectively and objectively, to warrant a report of the case. The end result cannot now be given, because treatment has not been concluded.

Local examination showed nothing of note in the anterior nares on either side. The posterior ends of both inferior turbinated bodies appeared as greyish-white masses, of irregular shape, compressible and nearly smooth. The left nostril was practically occluded by the enlargement on that side, which hung slightly backwards into the naso-pharynx. Both enlargements were bathed in an abundant, stringy mucus. The usual lump of greenish mucus and detritus was loosely adherent to the roof of the pharynx. The throat presented the picture of a post-diphtheritic paralysis. Muscular action related almost entirely to the right side, constrictors and elevators as well. The whole part was weak, thick and flabby, and pretty liberally covered with a thick, pasty secretion. Further down there was a considerable collection of mucus, at the base of the epiglottis, and in the pyriform sinuses on either side. This explained, in part, the patient's statement that he is always obliged to clear his throat before speaking. The larynx was quite normal in appearance and movement. At the conclusion of the examination, thus far, the patient voluntarily stated that it seemed as if he swallowed mostly with the right side. This was the stronger. Also, that particles of food, such as crumbs, frequently lodged behind his nose. The latter symptom is rarely absent in cases of faucial paresis, of whatever origin.

Unlike the first case, the half-dozen graduated bougies passed through the esophagus without hindrance, and with surprisingly little annoyance to the patient. They were used but twice. On the second occasion, the patient had purposely refrained from drinking to force onward a portion of food which had stuck at the usual place, which is his habit. This was done of his own accord. The bougie did not free the lodgment, but it was disposed of by subsequent drinking. The only explanation I could offer of this was habit, or on the principle of a cinder in the eye: it seems to be there long after its removal. If a diverticulum is suggested, I will give my objection to that. The right side of the throat was fairly sensitive to the faradic current. There was no sensation whatever on the left side until several times the strength of current was turned on; nor did the constrictor muscles respond until there was sensory disturbance. There was marked difference in the action of either half of the levator palati, and about the same degree of difference as to sensation and slight movement of the membrane applied to the wall of the pharynx. No inequality was noticed in the tongue, cheeks or roof of the mouth. Articulation was not perfect on certain words, and the lips were carefully tested, but without result. The patient whistled with the right side. He claimed to note a difference in the facial nerve, but I doubt this. An electrode introduced into the esophagus, to the length of three or four inches, was not noticed at all when the current was applied to the left side, whereas the patient nearly rose out of the chair when the opposite side was touched.

Before operating the parts within the nose previously mentioned, faradisation was repeated a few times as described and in the usual way upon the neck. I preferred, however, to postpone the systematic use of the current until the supposed sources of irritation had been removed and the parts readjusted in some measure. There was, nevertheless, rapid sensory and motor improvement on the paretic side. That this was not accompanied by a corresponding change in swallowing was a paradox to the patient. The explanation offered was, that a member of the body, as an arm, might readily be made to respond to any desired movement, even after a long period of rest; its movement might be quite as perfect as that of the well arm, yet if a weight were to be lifted a great difference in *muscular* capacity at once would be apparent.

It is significant that a much less amount of cocain than is generally required prepared the throat and naso-pharynx for operation. With the aluminum palate retractor in place (which I show because you may not all be familiar with it; which, because of its extreme lightness and flexibility, has reduced all naso-pharyngeal operating to exact manipulative technique), the tongue depressor being held by the patient, and the part illuminated with the rhinoscope, the enlarged posterior end of the inferior turbinated body on the right side was at once removed with the cold snare. Resting for a moment, the patient said he had a sensation as of a big hole somewhere behind his nose. On the other side the part was thoroughly destroyed with the galvano-cautery. The latter procedure for ablation of polypoid thickening is greatly to be preferred when practicable. A few patches of enlarged follicles on the wall of the pharynx were destroyed with the cautery point, as well as certain areas of thickened membrane, notably on the pillars of the fauces. Their further nutrition was cut off by interrupting the blood-vessels going to them. This last procedure is mainly directed toward the improvement of the condition known as "relaxed throat," present in this case. It is responsible, in whole or in part, for a long list of annoying symptoms, besides being a contributing factor in dysphagia, especially in singers and public speakers. I have never had opportunity to demonstrate my supposition that relaxation is thus helped by the formation of small areas or lines of scar tissue. If scar tissue does form, why would not it assist in rendering the part more tense? If its presence were only temporary, why would not it act in the same way while the part was adjusting itself to proper action, after the removal of some obstacle, such as intranasal obstruction? It is because of this teaching the part how to work properly when it can, after long-continued and forced misuse, that lessons in the use of the voice are so valuable and, in many cases, an indispensable adjunct to operative procedure.

The remainder of the treatment consisted at first in the daily application of the faradic current, within and without, alternating with painting certain thickened parts, which did not indicate the use of the cautery, with a solution of iodine and iodide of potash in glycerine; also the vaporisation of the upper air tract, through the nose, with that solution, combined with a solution of antiseptic substances in oil, by means of a multiple comminator. No medicine internally has been given thus far, and direction as to hygiene was found to be unnecessary.

The points in this case are, that the patient has been all along subjected to the effect of wrong impressions, which has undoubtedly affected his character in many ways, holding him back when his inclination was to go ahead. Further, if there is such a thing as reflex disturbance dependent upon intranasal irritation, this is such a case, and as such should have been recognised long ago. Thus damage would have been prevented, which at this late day may be remedied but is very probably not susceptible of complete cure.

After the many disappointing results we have had in the matter of reflex effect from intranasal disease, especially in hay-fever, often in cases of brilliant promise, the greatest degree of scepticism is warranted. It is, however, impossible to yield the relationship altogether. A case passed through my hands last summer, in which the whole nervous system seemed to explode within the nose, thence to distribute itself in fragments to functional disorder of various parts of the body. One or both nostrils would close suddenly and open again as suddenly after a longer or shorter time, or they would gradually become patent. Material which could swell was removed, as well as certain bony protuberances, and a deviation of the septum was straightened. The result was a delight to all concerned for about three months. An acute cold was then followed by a return of nearly the same old condition. The middle turbinated bodies were this time found to be the offending parts, alternately swelling into the space previously made or retreating from it, according as the patient was excited or calm, occupied in some absorbing way or unoccupied. Certain evidence is present in this case, in that the patient acquired the morphine habit for a short time, a year or so, previous to operation ; the habit was persisted in because she had no trouble with nasal obstruction pending the use of the drug. When the nose is closed, that person is utterly miserable. So long as a case of that nature exists ; so long as an epileptiform seizure can be produced by probing a certain area within the nose, an illustration of which occurred in my own practice not long since ; so long as cough, persisting for a year or more, and resisting all internal remedy, can be stopped almost at once by the removal of half-a-dozen large polypi from one nostril, the other being clear, which was also a recent most gratifying result ; so long as such an apparently simple matter as an erosion upon the septum can give rise to a long list of grievances against nature, represented, when fortunately brought to heal, by restoration to general health and the continuance of a college course, of which I can produce a witness of nearly a year's standing ; so long, in fact, as every vital process depends essentially upon proper breathing, which can be so readily interfered with by a multitude of mistakes in nasal anatomy, or acquisitions thereto, it would seem only fair to give the rhinologist a chance long before he usually gets it. Granted that he may do incalculable harm. The same is true of specialists in other departments. And it is a question if the harm done by the rhinologist is not more often apparent than real. The slightest thing wrong with the nose or throat is noticed and located at once. A great deal may be the matter with the uterus, for example, both before and subsequent to operation, hence with the entire body, yet the patient does not relate her malady especially to that organ. The same may be said of the stomach and bowels, and their many attempted curative measures, if so be the patient has a little rheumatism to absorb his attention.

There are several other reflex disturbances in the pharynx and mouth accredited to intranasal disease, such as hyperesthesia, paresthesia or imaginary foreign body, pain in the throat, paresis of the palate, cases of which are probably cases of chorea. Violent and distressing hiccough has been reported. Salivation, herpes and toothache have been described and noted as cured by leading foreign and American authorities. I have had cases under nearly all these headings, some of which I have been content to accept as proved. Next to hyperesthesia, dysphagia is the commonest reflex disturbance from intranasal disease which can be benefited or cured. Clinical proof is what is wanted, and a multitude of cases is often confusing and damaging and of less value than two or three which are positively convincing. Clinical fact should, however, be in agreement with theory. I shall, therefore, conclude this sketch with an attempt to show, theoretically, how intranasal disease and dysphagia can be connected. If that can be done, it stands to reason that other reflex disturbances, supposedly from a similar cause, can be equally well explained.

Let me remind you of the "reflex arc." It consists in (1) an afferent sensory nerve, (2) an efferent motor nerve, and (3) a center of nerve cells or fibers in the spinal cord connecting the two. A sensory impulse can find its expression in a given area as a vascular change, due to its transmission as a vaso-motor impulse interfering with the nerve control of the arteries in that area. For instance, a local morbid condition is followed by an impression to the nerve center of its existence, and this, in turn, appears as transmitted force to another part, remote or near, resulting in some pathological condition or other subjective evidence. The medulla controls the nerve supply of the blood-vessels and is the coördinating center of all reflexes which have to do with the support of vital functions. These functions can be disturbed by irritation of any sensory nerve in the body. The spheno-palatine ganglion has been the point of experimental attack to explain reflex disturbances from intranasal disease. The trigeminus nerve has its origin in this ganglion, and it is this nerve which controls the secretion and probably the nutrition of the nose. We have but to recollect the extent of distribution of this nerve, its anastomoses and ganglionic connections ; also that it regulates the most intricate and delicately sensitive portion of the whole respiratory tract, to appreciate what abundant opportunity is offered within the nose for reflex disturbances. In the matter of reflex dysphagia from nasal disease, the course of transmission appears to be by way of the sensory root of the trigeminus in its connection at that point with the Gasserian ganglion. The next link is through the carotid plexus of the superior cervical ganglion of the sympathetic and the pneumogastric, whose fibers are distributed to the pharynx and esophagus. The naso-pharynx and pharynx, while partaking of the general structural character of the upper respiratory tract, have to do more especially with deglutition. There are the tonsils on either side out of the way of injury, and the glands in the vault of the pharynx, similarly protected, supposedly supplying a good deal of secretion as a factor in lubricating a bolus of food. When the action of these glands is interfered with, deglutition is likewise affected. Nothing disturbs their action to any such extent as impaired or lost innervation of the constrictor muscles of the pharynx. These muscles have their nerve supply from the pharyngeal plexus, which is formed by the largest, or carotid branch of the ninth pair, uniting with the pharyngeal branch of the sympathetic. Without considering details of mechanism, the chain of connection from intranasal irritation to dysphagia is, therefore, complete.

The next question relates to the most frequent cause of starting of a nasal reflex. In the majority of cases this is due emphatically to turgescence of the inferior turbinated bodies. The vascular tissue of which they are principally composed, notably veins or venous sinuses, is erectile. When this becomes engorged with blood, tension of the mucous membrane ensues, which represents a source of irritation to the sensory nerve filaments, and a reflex train is set in motion. Any part within the nose which is capable of receiving an undue flux of blood, will act in the same way. An instance of this is found in the soft oval swellings so frequently seen at the lower and extreme posterior portion of the septum. I recently saw a case markedly illustrative of the last condition. Pressure with the probe caused severe pain across the temple, along the side of the head and downward into the jaw. Whenever the parts were swollen, headache was invariably present. As soon as the effect of the cautery had subsided, the pain disappeared and has not returned. A hyperemic rather than a hypertrophic condition is, therefore, more favorable as a starting point. For this reason polypoid thickening is an exceedingly fertile soil for the development of reflexes, and this is the nature of the enlargements noted in the second case. Extreme congestion represents a vaso-motor paresis, and is most frequently found in persons of a neurotic habit. Hypertrophic changes, on the other hand, are often manifestations of purely local disease. It is, however, often not the initial lesion which causes the disturbance, but the paretic condition to which it gives rise. Constant severe vascular disturbance sometimes produces a hyperesthetic condition of the nerve centers, and reflex excitability is thereby greatly heightened. This explains, probably, the minimum amount of benefit from treatment of neglected cases of long standing—in which category my second case will undoubtedly fall.

Engorgement is not by any means necessary for reflex action. I have a case at present in which reflex headache was disposed of by the fortunate healing of an erosion upon the septum anteriorly at a vulnerable spot. I cannot agree, however, that pure atrophic nasal disease is causative of reflex excitation ; nor can I endorse Gottstein's opinion that vaso-motor neurosis in these cases is due to the neurosis itself, and that the result of cauterisation in disposing of the reflex is a matter of luck or is due to revulsive action.

Finally, a few words upon dysphagia, *per se*, and as to differential diagnosis. It is caused by disease or injury of the fauces or esophagus, or it is reflex. In addition to many local causes listed in the text-books one is often omitted—namely, enlarged glands at the base of the tongue. I would note also that auscultation of the esophagus frequently gives valuable diagnostic information and is not generally practised.

Differential diagnosis relates to (1) spasm, (2) chronic esophagitis, (3) paresis, (4) malignant or specific disease. The principal points to note are these : In spasm the sound or bougie passes with great difficulty, whereas in the other conditions (unless there is positive stenosis) these instruments pass more readily than in health, even without a tendency to gagging. Dysphagia is present in all, but it is intermittent and of variable intensity in spasm, constant and without special variation in other diseases. Regurgitation is a symptom common to all. It is, however, much more frequent in spasm. In this condition also solids as well as liquids are expelled, usually immediately and forcibly ; in organic disease liquids or fluids are more readily swallowed than solids, and the former are more often expelled. Other diagnostic points of value are that in spasm the symptoms develop suddenly, pain is rarely present, and there is never a frothy admixture in regurgitated material. In spasm the patient is rarely emaciated though weak, nor is he especially depressed.

It is not necessary to consider the points of difference between various forms of organic disease further than to note that paresis of a myopathic nature is not infrequently a sequela of acute febrile disease, as well as from disturbed innervation and blood supply in consequence of intranasal disease. Hysteria rarely leads to paresis of the pharyngo-esophageal musculature, but is manifested as a spasm. In local paresis development of dysphagia is very gradual. Morrell Mackenzie refers to many cases in his practice which lasted from ten to twenty years. For emphasis I refer again, and separately, to auscultation of the esophagus. In paresis a mumbled, gurgling sound is heard, whereas in spasm the sound is sharp and clicking, heard now in one place, now in another.

Prognosis in simple local paresis, as from muscular weakness, is always favorable. Improvement rarely fails and cure results in many cases.

When artificial feeding is necessary and can be given by the mouth, a bottle with a tube and bulb attachment, as suggested by Bryson Delavan, of New York, will be found to be an excellent device.

For internal medication as against neurotic diathesis, a combination of the extract of belladonna with the oxide of zinc, in pill or tablet, in the proportion of a quarter of a grain of the former to half a grain of the latter, three or four times daily, has given better results, in my experience, than strychnia, nux vomica or the various proprietary nerve tonics.

I would also refer again to hyperesthesia of the pharynx as the most common of the reflex disturbances of intranasal disease, and ask a trial of a spray of resorcin to assist in controlling that condition.

21 West North Street.

